# Potential of Exosomal microRNA-200b as Liquid Biopsy Marker in Pancreatic Ductal Adenocarcinoma

**DOI:** 10.3390/cancers12010197

**Published:** 2020-01-13

**Authors:** Moritz Reese, Isabelle Flammang, Zixuan Yang, Sameer A. Dhayat

**Affiliations:** Department of General, Visceral and Transplantation Surgery, University Hospital Muenster, Albert-Schweitzer-Campus 1 (W1), 48149 Muenster, Germany; m_rees04@uni-muenster.de (M.R.); isabelle.flammang@ukmuenster.de (I.F.); yzxbcchina@uni-muenster.de (Z.Y.)

**Keywords:** pancreatic ductal adenocarcinoma, microRNA, liquid biopsy, exosomes, epithelial cell adhesion molecule

## Abstract

Pancreatic ductal adenocarcinoma (PDAC) is a highly malignant tumor entity, characterized by rapid disease progression, early metastatic dissemination, and late diagnosis at advanced tumor stages. Recently, we explored the clinical impact of several microRNAs (miR) associated with proliferation, epithelial-to-mesenchymal transition (EMT), and chemoresistance in tissue and blood serum specimens of PDAC patients. Here, we evaluated the potential of these miRs as diagnostic and prognostic biomarkers in PDAC in serum exosomes and their respective EpCAM-positive (epithelial cell adhesion molecule) subset. Expression analysis by RT-qRT-PCR (real-time quantitative reverse transcription polymerase chain reaction) revealed an overexpression of miR-200b and miR-200c in serum exosomes of PDAC patients as compared to healthy controls (*p* < 0.001; *p* = 0.024) and patients with chronic pancreatitis (*p* = 0.005; *p* = 0.19). Receiver operating characteristic (ROC) curve analysis showed that a biomarker panel consisting of miR-200b and miR-200c from total and EpCAM-positive serum exosomes enhanced the diagnostic accuracy of carbohydrate antigen 19-9 (CA.19-9) to 97% (*p* < 0.0001). Univariate survival analysis revealed a correlation between shorter overall survival (OS) and high expression of miR-200c in total serum exosomes (*p* = 0.038) and miR-200b in EpCAM-positive serum exosomes (*p* = 0.032), whereas EpCAM exosomal miR-200b was also indicative of shorter OS in the subgroup of patients treated with curative intent (*p* = 0.013). Multivariate survival analysis showed that miR-200b derived from EpCAM-positive serum exosomes might serve as an independent prognostic factor in PDAC (*p* = 0.044). Our findings indicate a potential role of exosomal miR-200 as diagnostic and prognostic liquid biopsy marker in PDAC and call for validation in a larger, multicenter setting.

## 1. Introduction

Pancreatic ductal adenocarcinoma (PDAC) is a highly lethal tumor entity. Although being only the 11th most common malignancy in the United States, it ranks third in terms of cancer-related deaths and will account for approximately 45,750 deaths in 2019 with a dismal five-year survival rate below 10% [1]. The only potentially curative treatment option is surgical resection, however, the majority of patients are diagnosed at unresectable stages. Medication-based treatment options for PDAC are limited, and FOLFIRINOX (folinic acid, fluorouracil, irinotecan, oxaliplatin) and gemcitabine-based therapeutic regimens are standards of care in both the (neo-)adjuvant and palliative setting [2]. Response rates, however, are low, and this is attributed to PDAC being a highly chemoresistant tumor type. As opposed to other malignancies, the advances made over recent years in treating PDAC have been marginal. Targeted molecular therapies and immunotherapies that have revolutionized the treatment of multiple types of cancer have been mostly unsuccessful in PDAC [3,4,5,6,7,8,9]. Hence, diagnosing PDAC at early, potentially resectable stages is a key strategy in improving patients’ prognosis.

Carbohydrate antigen 19-9 (CA.19-9) is currently the only tumor marker approved by the Food and Drug Administration (FDA) in the management of PDAC. Despite being utilized in follow-up and aftercare, it is relatively unspecific and not all PDAC patients show elevated blood levels of CA.19-9, rendering it ineffective (1) as a screening tool for early detection of pancreatic neoplasms and (2) as a decisive marker for the differentiation of early-stage malignant and non-malignant conditions of the pancreas. In the course of finding more reliable biomarkers, microRNAs (miRs) have raised the attention of medical research. MiRs are well-preserved, small non-coding RNAs that are about 22 nucleotides long and play a critical regulatory role in post-transcriptional inhibition of gene expression [10]. They have been attributed tumor-suppressive as well as oncogenic functions in multiple tumor entities and we have previously reported on their diagnostic and prognostic potential, as well as their influence on chemoresistance and epithelial-to-mesenchymal transition (EMT) in PDAC [11,12,13,14,15,16]. It is not without reason that miRs are of particular interest to researchers—circulating plasma or serum miRs can be stored at −80 °C for many years and are resistant to several freeze-thaw cycles and incubation at room temperature for over 24 h [17,18]. Interestingly, one miR targets many messenger RNAs, which could potentially translate into modulating multiple pathologically dysregulated genetic pathways with a single molecule. This pleiotropic nature of miRs makes them attractive as drug targets for PDAC, a multifactorial disease lacking effective treatment options.

Mounting evidence is pointing towards the fact that deregulation of exosomal miRs plays a role in many types of diseases, especially cancer [19]. Exosomes are small membrane vesicles of endosomal origin with a diameter of approximately 30–100 nm [20]. They are released by multiple types of cells including cancer cells into the extracellular environment and they have been shown to play an important role in intercellular communication, carrying proteins, RNA, and DNA [21,22]. In the bloodstream, exosomes from malignant tissue are diluted with exosomes secreted by healthy tissue. Hence, considerable effort has been devoted to finding proteins that are more selectively expressed on tumor-derived exosomes. It has been reported that this subset of exosomes could be isolated using anti-EpCAM (epithelial cell adhesion molecule) antibodies in combination with magnetic beads [23]. As such, EpCAM-positive exosomes have been shown to be more specifically released by epithelial tumors including PDAC [24,25,26].

In this study, we investigated the clinical relevance of a panel of miRs in serum exosomes of PDAC patients. Among those miRs studied, miR-21 especially is a well-known oncogenic miR involved in tumorigenesis, progression, and therapy resistance of cancer. In non-small cell lung cancer, miR-21 increases K-ras-dependent (Kirsten-rat sarcoma viral oncogene homolog) tumor formation, whereas in colorectal cancer, expression of miR-21 was reported to be inversely correlated with expression of tumor suppressor gene PDCD4 (programmed cell death protein 4), and in PDAC modulates gemcitabine resistance and correlates with overall survival (OS) [27,28,29]. Similarly, miR-155 and miR-1246 have been shown to contribute to gemcitabine resistance and were identified as potential prognostic markers in PDAC [30,31]. MiR-125b and members of the miR-99 family consisting of miRs 99a, 99b, and 100 have also been attributed oncogenic functions, regulating PDAC progression and serving as prognostic markers and predictors of chemo-responsiveness [13,32]. Conversely, miRs 34a, 148a, 200a, 200b, and 200c function as tumor-suppressive miRs in PDAC with prognostic impact that predominantly inhibit EMT, a process closely related to metastatic dissemination [14,33,34,35,36].

## 2. Results

### 2.1. Clinicopathologic Patient Data

A total of 89 patients were included in this study, thereof 22 healthy patients, 11 patients with chronic pancreatitis, and 56 patients with PDAC. PDAC patients are subdivided into 18 UICC (Union for International Cancer Control) stage II, 22 UICC stage III, and 16 UICC stage IV according to the eighth edition of the TNM (Tumor, Node, Metastasis) classification of malignant tumors. Clinicopathologic data of all patients are shown in Table 1. No significant differences were detected between the different study groups for any characteristic, except for pre-surgical pancreatitis (*p* < 0.001) and pre-surgical blood serum level of CA.19-9 (*p* = 0.007). The distribution of histopathologic characteristics across UICC tumor stages of PDAC patients is summarized in Table S1. Moreover, log-rank subgroup analysis of PDAC patients revealed significant differences in median OS with regard to UICC stage (*p* = 0.013), metastasis (*p* = 0.008), type of surgery (*p* = 0.006), and administration of chemotherapy (*p* < 0.001) (Table 2). No significant differences in median OS could be detected for tumor grading (*p* = 0.252), lymphatic invasion (*p* = 0.995), perineural invasion (*p* = 0.142), vene invasion (*p* = 0.215), and resection margin (*p* = 0.533).

### 2.2. Expression Analysis of a microRNA Panel in Serum Exosomes

**Figure 1:**
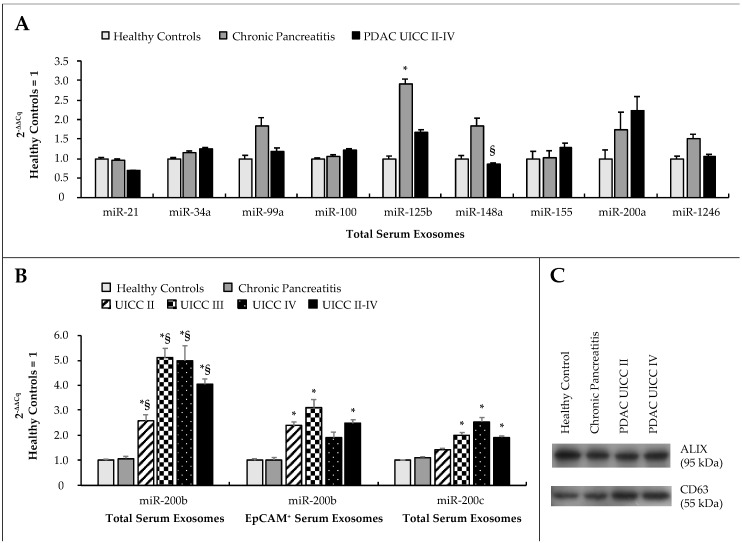
(**A**) Expression of a panel of miRs in circulating serum exosomes and (**B**) expression of miR-200b in total and EpCAM (epithelial cell adhesion molecule)-positive serum exosomes and of miR-200c in total serum exosomes. Data were analyzed by RT-qRT-PCR and plotted as 2^−ΔΔCq^ ± standard error of the mean (SEM), relative to healthy controls. Statistical significance (*p* ≤ 0.05, Kruskal–Wallis test) is indicated relative to healthy controls (*) and chronic pancreatitis (§). (**C**) Western blot for exosomal markers ALIX (apoptosis-linked gene 2—interacting protein X) and CD63 (cluster of differentiation 63) in exosomes isolated from patients’ blood serum specimens.

On the basis of our previous work and a review of the literature, we selected and quantified a panel of 11 miRs consisting of miR-21, -34a, -99a, -100, -125b, -148a, -155, -200a, -200b, -200c, and -1246 by RT-qRT-PCR in circulating exosomes derived preoperatively from patients’ blood serum samples (Figure 1). Exosomes were isolated from patients’ blood serum samples by differential centrifugation and verified by western blotting for exosomal markers ALIX (apoptosis-linked gene 2—interacting protein X) and CD63 (cluster of differentiation 63) (Figure S1). Expression of miR-200b and miR-200c was significantly deregulated in serum exosomes of PDAC patients compared to healthy patients (*p* < 0.001; *p* = 0.024) and patients with chronic pancreatitis (CP) (*p* = 0.005; *p* = 0.19). There were no significant differences in expression between healthy patients and patients with malignant disease for any other exosomal miR. MiR-125b was significantly deregulated in patients with CP compared to healthy controls (*p* = 0.008), and expression of miR-148a was significantly higher in patients with CP as compared to patients with PDAC (*p* = 0.008). In view of these expression data, miRs 200b and 200c in particular were analyzed in total serum exosomes and additionally in the subfraction of serum exosomes positive for EpCAM.

### 2.3. Differential Expression Analysis of miR-200b and miR-200c in Circulating Serum Exosomes

Exosomal expression of miR-200b and miR-200c was quantified by RT-qRT-PCR in 89 patients, consisting of 22 healthy controls, 11 patients with CP, and 56 patients with PDAC, thereof 18 patients with PDAC UICC stage II, 22 patients with PDAC UICC stage III, and 16 patients with PDAC UICC stage IV (Table S2). Exosomal miR-200b was significantly upregulated in total serum exosomes across all tumor stages and when taking together all PDAC compared to healthy controls (UICC II: 2^−ΔΔCq^ = 2.57, *p* = 0.037, *r* = 0.33; UICC III: 2^−ΔΔCq^ = 5.1, *p* < 0.001, *r* = 0.61; UICC IV: 2^−ΔΔCq^ = 4.99, *p* = 0.001, *r* = 0.53; UICC II-IV: 2^−ΔΔCq^ = 4.04, *p* < 0.001, *r* = 0.45) as well as chronic pancreatitis (UICC II: 2^−ΔΔCq^ = 2.38, *p* = 0.001, *r* = 0.29; UICC III: 2^−ΔΔCq^ = 4.72, *p* = 0.002, *r* = 0.55; UICC IV: 2^−ΔΔCq^ = 4.61, *p* = 0.011, *r* = 0.50; UICC II-IV: 2^−ΔΔCq^ = 3.73, *p* = 0.005, *r* = 0.35). Looking at the subgroup of EpCAM-positive serum exosomes, miR-200b was deregulated in non-metastasized UICC tumor stages II (2^−ΔΔCq^ = 2.38, *p* = 0.02, *r* = 0.37) and III (2^−ΔΔCq^ = 3.11, *p* = 0.013, *r* = 0.38) and when comparing all PDAC to healthy patients (2^−ΔΔCq^ = 2.48, *p* = 0.008, *r* = 0.30). There were no significant differences for any subgroup of PDAC when compared to chronic pancreatitis. In total serum exosomes, miR-200c was upregulated in patients with UICC stages III (2^−ΔΔCq^ = 2.02, *p* = 0.046, *r* = 0.31) and IV (2^−ΔΔCq^ = 2.55, *p* = 0.022, *r* = 0.38) compared to healthy controls and when comparing all PDAC with healthy patients (2^−ΔΔCq^ = 1.92, *p* = 0.024, *r* = 0.26). No significant differences in relative exosomal expression of miR-200c could be detected for any pairwise comparison in the subfraction of EpCAM-positive serum exosomes.

### 2.4. Diagnostic Analysis of Circulating Exosomal miR-200b and miR-200c

**Figure 2:**
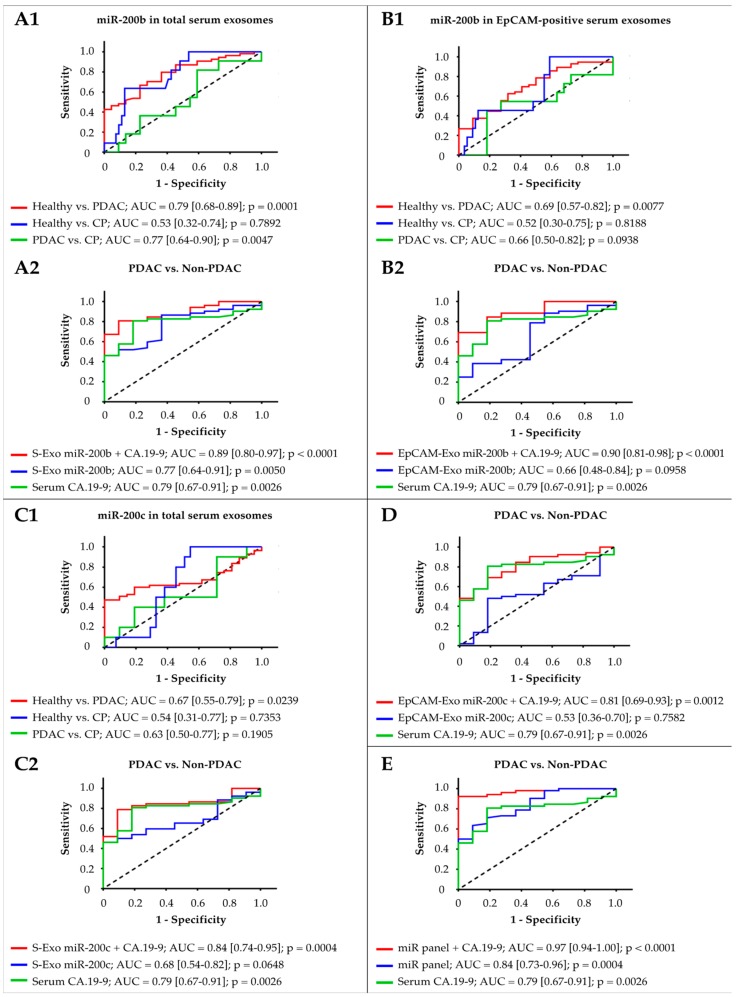
Receiver operating characteristic curve analysis for discrimination between (1) healthy, chronic pancreatitis, and PDAC, as well as (2) PDAC and non-PDAC, in comparison to and in combination with CA.19-9. (**A**) MiR-200b from total serum exosomes, (**B**) miR-200b from EpCAM-positive serum exosomes, (**C**) miR-200c from total serum exosomes, and (**D**) miR-200c from EpCAM-positive serum exosomes. (**E**) Diagnostic potential of a biomarker panel consisting of all four exosome fractions in comparison to and in combination with CA.19-9. AUC, area under the ROC curve.

Calculating the area under the ROC (receiver operating characteristic) curve (AUC), exosomal miR-200b could differentiate PDAC patients from healthy controls (AUC of 0.79; *p* = 0.0001) and from patients with chronic pancreatitis (AUC of 0.77; *p* = 0.0047) with good accuracy. Its ability to differentiate PDAC cases from non-PDAC cases (AUC of 0.77; *p* = 0.005) was similar to that of tumor marker CA-19.9 (AUC of 0.79, *p* = 0.0026). Combining serum exosomal miR-200b and CA-19.9 allowed for excellent discrimination between PDAC and non-PDAC (AUC of 0.89; *p* < 0.0001; sensitivity: 0.81, specificity: 0.91, likelihood ratio: 8.9). MiR-200b derived from EpCAM-positive serum exosomes did not reach the diagnostic potential of CA-19.9 in differentiating PDAC from non-PDAC (AUC of 0.66; *p* = 0.0958 vs. AUC of 0.79; *p* = 0.0026) but it was suitable to distinguish PDAC from healthy patients (AUC of 0.69; *p* = 0.0077) and in combination with CA.19-9 was an excellent discriminator between PDAC and non-PDAC (AUC of 0.90; *p* < 0.0001; sensitivity: 0.69, specificity: 1.00). MiR-200c from total serum exosomes could differentiate PDAC from healthy controls (AUC of 0.67; *p* = 0.0239) with fair accuracy, but not from chronic pancreatitis. Both miR-200c from total serum exosomes and EpCAM-positive serum exosomes in combination with CA-19.9 enhanced the tumor marker’s ability to distinguish between PDAC and non-PDAC. For total serum exosomes, AUC could be improved to 0.84 (*p* = 0.0004; sensitivity: 0.79, specificity: 0.91, likelihood ratio: 8.7). For EpCAM-positive serum exosomes, AUC could be improved to 0.81 (*p* = 0.0012; sensitivity: 0.69, specificity: 0.82, likelihood ratio: 3.8). Combining all four miR fractions with CA.19-9 entailed an outstanding diagnostic accuracy of 97% (*p* < 0.0001). PDAC could then be distinguished from non-PDAC with a sensitivity of 0.92 and specificity of 1 (Figure 2). In terms of distinguishing between subgroups of PDAC UICC tumor stages, the previously mentioned biomarker panel could differentiate between UICC tumor stages II and IV with fair accuracy (AUC of 0.75; *p* = 0.0129) but not between UICC stages II and III, or III and IV (Figure S2).

### 2.5. Survival Analysis of Circulating Exosomal miR-200b and miR-200c

**Figure 3:**
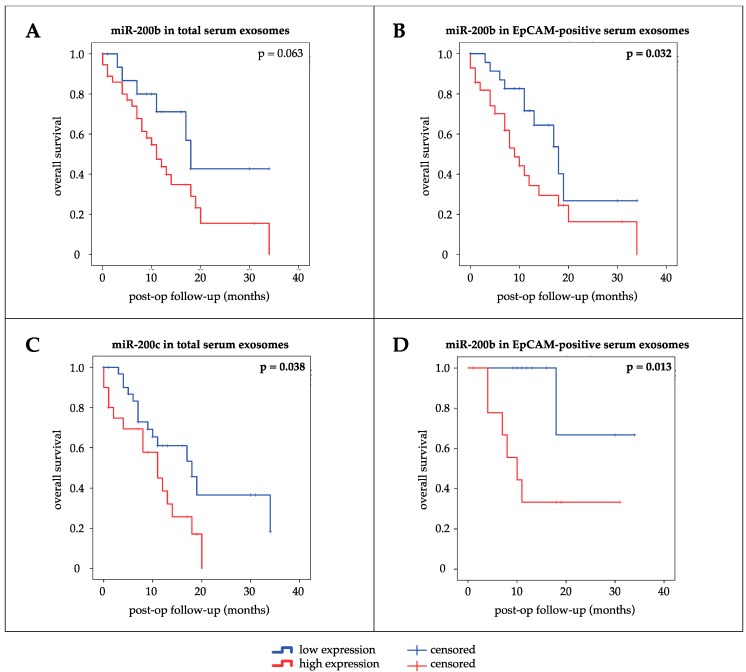
Kaplan–Meier curves for univariate survival analysis. Bold values indicate significance (*p* ≤ 0.05, log-rank test). Prognostic impact on overall survival of all PDAC patients of (**A**) miR-200b in total serum exosomes, (**B**) miR-200b in EpCAM-positive serum exosomes, (**C**) miR-200c in total serum exosomes. (**D**) Prognostic impact of miR-200b in EpCAM-positive serum exosomes on overall survival of patients treated with curative intent.

The prognostic influence of exosomal expression of miRs 200b and 200c on OS and recurrence-free survival (RFS) was assessed by univariate survival analysis using Kaplan–Meier curves and log-rank test (Figure 3). High expression of miR-200b in EpCAM-positive serum exosomes (*p* = 0.032, median OS 9 months (high) vs. 18 months (low)) and miR-200c in total serum exosomes (*p* = 0.038, median OS 11 months (high) vs. 18 months (low)) were associated with shorter OS of patients. Though not significant, high expression of miR-200b in total serum exosomes seemed to correlate with shorter OS as well (*p* = 0.063). High expression of EpCAM-positive serum exosomal miR-200b was also associated with shorter OS in the subgroup of PDAC patients treated with curative intent (*p* = 0.013, median OS 10 months (high) vs. not reached (NR) (low)). No statistically significant differences in RFS could be detected between groups of high and low expression for any exosomal miR (miR-200b serum exosomes: *p* = 0.306; miR-200b EpCAM^+^ serum exosomes: *p* = 0.187; miR-200c serum exosomes: *p* = 0.084; miR-200c EpCAM^+^ serum exosomes: *p* = 0.093). A Cox proportional-hazards model was applied to identify statistically significant differences between subgroups of dichotomized factors with regard to OS of PDAC patients. Univariate survival analysis revealed UICC stage II (*p* = 0.010), absence of distant metastasis (*p* = 0.013), application of chemotherapy (*p* = 0.002), low expression of miR-200b in EpCAM-positive serum exosomes (*p* = 0.040), and low expression of serum exosomal miR-200c (*p* = 0.046) as potentially favorable prognostic factors. After adjusting for confounding factors, multivariate survival analysis identified application of chemotherapy (*p* = 0.001) and low expression of miR-200b in EpCAM-positive serum exosomes (*p* = 0.044) to be independent prognostic factors in PDAC favoring prolonged OS of patients (Table 3).

## 3. Discussion

Despite multiple breakthroughs achieved in oncologic research over the last decades, the advances made in treating PDAC have been disappointing. If resected successfully, however, overall five-year survival rates of PDAC patients can be increased to 15–40% [37]. Taking into consideration that approximately 80% of patients initially present with unresectable disease, a promising approach to substantially improve their prognosis seems to be identification of PDAC at an early tumor stage. As for those patients ineligible for surgery, overcoming chemoresistance is key in halting rapid disease progression. At present, however, no serum-based or other clinical biomarker has a sufficient sensitivity or specificity for use in clinical routine, which could translate into early diagnosis and monitoring of therapy-response in PDAC patients [38]. Over the past years, miRs have increasingly been put into the spotlight of oncologic research as promising liquid biopsy markers, and first multicenter studies have been initiated to validate the applicability of miRs in the clinical setting. Recently, a blood-derived miR diagnostic test was shown to considerably enhance the value of low-dose computed tomography in lung cancer screening in a large prospective randomized trial enrolling more than 4000 patients at risk. The combination of low-dose computed tomography and miR blood test was highly predictive for lung cancer with a four-year incidence of 20.1% for patients with double-positive screening results, as compared to 3.8% and 0.6% for single-negative and double-negative screening results, respectively [39].

In the present study, we could demonstrate that serum exosomal miR-200 has diagnostic and prognostic potential in PDAC. Members of the miR-200 family have been implied diagnostic value in various malignancies, including gastric cancer, hepatocellular carcinoma, and ovarian cancer, and recently we emphasized the diagnostic and prognostic potential of a panel of EMT-related miRs including miR-200b and miR-200c in tissue and serum specimens of PDAC patients [14,40,41,42]. Here, circulating exosomal miR-200c (AUC = 0.70) did not reach the previously reported diagnostic accuracy of miR-200c derived from tissue (AUC = 0.84) or blood serum (AUC = 0.78) in differentiating between PDAC and non-PDAC. In contrast, the diagnostic accuracy of serum exosomal miR-200b (AUC = 0.79) was similar to that of miR-200b derived from tissue (AUC = 0.72) or blood serum (AUC = 0.79). Previous trials have shown that the diagnostic significance of single exosome-derived miRs can be limited. However, combinations of circulating miRs, particularly exosome-derived miRs, may serve as powerful minimally invasive diagnostic biomarkers that are superior to or can potentially enhance the sensitivity and specificity of established tumor markers. This was shown in several malignancies, including lung cancer, ovarian cancer, and PDAC [42,43,44,45]. Taking into account that tumor-derived exosomes are diluted in the bloodstream with a large number of vesicles secreted by healthy tissue, researchers have identified proteins such as EpCAM that were reported to be more selectively expressed on tumor-associated vesicles of PDAC cells [24,25]. In accordance with these findings, we could show that combining CA.19-9 with serum exosomal miR-200b and miR-200c in total as well as EpCAM-positive serum exosomes improved the tumor marker’s diagnostic accuracy by 18%, yielding an AUC of 0.97, sensitivity of 92%, and specificity of 100%. However, the biomarker panel’s ability to differentiate between subgroups of PDAC UICC stages was restricted, possibly due to the limited study power. Evaluating its suitability for distinguishing early from late-stage PDAC hence remains an interesting prospect for future investigation.

Multivariate survival analysis identified miR-200b derived from EpCAM-positive serum exosomes as a potentially independent prognostic marker in PDAC. MiR-200b and miR-200c derived from circulating exosomes have already been shown to possess diagnostic and prognostic value in ovarian cancer, lung cancer, colon cancer, prostate cancer, and melanoma [42,45,46,47,48]. To our knowledge, our study is the first to present the diagnostic and prognostic value of miR-200b and miR-200c in circulating serum exosomes and their respective EpCAM-positive subfraction in PDAC. Among the other miRs studied, miR-21, miR-155, and miR-1246 have previously been reported to be deregulated in circulating exosomes of PDAC patients. These results could not be verified in our cohort; however, we hypothesize that this might be due to the small number of patients enrolled in these studies [49,50,51].

As members of the miR-200 family, miR-200b and miR-200c are among the best studied miRs in oncology. Although it is widely accepted that the miR-200 family acts as a tumor suppressor and regulator of EMT in multiple tumor types, there have been discrepancies with regard to its expression profile in different clinical specimens. More specifically, miR-200 seems to be upregulated in serum and circulating exosomes but has mostly been described as being downregulated in tumor tissue [14,42,52]. One hypothesis trying to explain this phenomenon is that tumors might actively shed tumor-suppressive miRs from their cells in order to facilitate the induction of metastatic dissemination. Another explanatory approach is based on the assumption that elevated levels of circulating and exosome-derived miR-200 could be ascribed to circulating tumor cells undergoing the final steps of the metastatic cascade. In breast cancer, metastatic cells were shown to overexpress and secrete miR-200 in extracellular vesicles, which contributed substantially to the ability of colonizing distant organs [53]. Either way, our results are supported by Lee et al. and their meta-analysis on the prognostic value of miR-200 in cancer. It revealed that low expression of miR-200 in cancer tissue correlates with prolonged OS, whereas high expression of circulating miR-200 correlates with shorter OS of patients, a clinical observation that remains to be untangled at the molecular level [54].

It is believed that in recipient cells, horizontal transfer of tumor-derived exosomal miRs can induce processes such as tumor progression and metastatic dissemination [55]. This makes miRs attractive candidates for diagnostic and therapeutic purposes in clinical applications. In 2019, for example, the nomination of the anti-miR-10b oligonucleotide RGLS5579 for the treatment of glioblastoma multiforme was announced. In an orthotopic glioblastoma multiforme animal model, RGLS5579 as monotherapy and in combination with temozolomide resulted in a significant increase in median OS of 18% and 159% versus control, respectively [56]. To our knowledge, Cobomarsen (MRG-106) and TargomiR-1 currently are the only two miR therapeutics being actively investigated in oncologic indications in the clinical setting. Cobomarsen is a miR-155 inhibitor being tested for cutaneous T cell lymphoma in a randomized phase II trial enrolling 126 participants (NCT03713320). TargomiR-1, a miR-16 mimic miR, successfully completed a phase I trial in malignant pleura mesothelioma with a disease control rate of 73% [57]. Further clinical trials on TargomiR-1 are expected to be initiated in the future.

Although no other miR drug candidates have been entered into clinicaltrials.gov database, a large number of registered clinical studies have been initiated for miRs as biomarkers [58]. These include observational studies exploring the impact of miRs as predictors of chemo-responsiveness in gastric (NCT03253107) and colorectal cancer (NCT02635087, NCT02466113) as well as diagnostic and prognostic liquid biopsies in pancreatic (NCT03432624), endometrial, and ovarian cancer (NCT03776630). Similarly, miRs derived from extracellular vesicles are investigated as diagnostic and prognostic biomarkers in prostate (NCT03694483, NCT03911999) and ovarian cancer (NCT03738319).

Understanding of the biology of specific exosomal miRs may be an essential step towards an improved diagnostic and personalized therapy in PDAC. Therefore, further adequately powered studies including early PDAC stages and its premalignant precursors are required to achieve the goal of developing exosome- and miR-based cancer biomarkers and therapeutic strategies.

## 4. Materials and Methods

### 4.1. Patients

All patients enrolled in this study were recruited between 2015 and 2018 by the Department of General, Visceral and Transplantation Surgery of the University Hospital Muenster. Ethical approval and informed written consent for the collection of blood serum, clinicopathological characteristics, and follow-up data were obtained from the ethics committee of the University of Muenster (reference numbers 1IXHai/11.8.2011 and 2016-074-f-S) and from every respective patient. The study was conducted in accordance with the Declaration of Helsinki and patients with immunosuppression and neoadjuvant chemo- or radiotherapy were excluded to eliminate irregularities with regard to miR expression. Patients with suspicion of resectable PDAC underwent pylorus-preserving pancreaticoduodenectomy (Traverso-Longmire procedure) or left-sided pancreatic resection depending on the location of the primary tumor, followed by approved adjuvant therapy. Excisional tumor biopsy was performed in advanced inoperable PDAC UICC stages III-IV for diagnostic reasons. PDAC patients were classified according to the eighth edition of the UICC TNM classification. Patients recruited prior to the initiation of the eighth edition of the UICC TNM classification were restaged for the purpose of this study. The primary endpoints of this study were overall and recurrence-free survival.

### 4.2. Collection of Whole Blood, Blood Serum Exosomes, and EpCAM-Positive Exosomes

Whole blood samples were collected from each patient before initiation of therapy, allowed to clot for 30 to 60 min and centrifuged at 2600× *g* for 10 min (Megafuge 1.0 R; Heraeus, Hanau, Germany). Serum supernatants were centrifuged at 12,100× *g* for 10 min (Minispin; Eppendorf, Hamburg, Germany) and stored at −80 °C. For isolation of total serum exosomes, samples were centrifuged at 16,000× *g* for 20 min (Biofuge 28RS, Heraeus), the supernatant was diluted with D-PBS (Dulbecco’s phosphate-buffered saline; Sigma-Aldrich, St. Louis, MO, USA) and centrifuged at 100,000× *g* and 4 °C for 3 h (Sorvall WX Ultra 80 ultracentrifuge, TH641 swinging bucket rotor; Thermo Fisher Scientific, Waltham, MA, USA). Thereafter, the supernatant was aspired and the leftover pellet resuspended in D-PBS. Samples were centrifuged at 100,000× *g* and 4 °C for another 3 h, the supernatant was again discarded, and the remaining pellet was resuspended in 100 µL D-PBS and stored at −80 °C. EpCAM-positive serum exosomes were isolated from total serum exosome samples in an immunoaffinty-based enrichment process using EpCAM-specific magnetic Dynabeads (Exosome-Human EpCAM Isolation Reagent; Thermo Fisher Scientific, Waltham, MA, USA) according to the manufacturer’s standard protocol. In short, pre-enriched serum exosome samples were incubated with magnetic beads and isolation buffer overnight at 4 °C in an overhead rotator (Bio RS-24 mini rotator; Biosan, Riga, Latvia). Samples were washed with 1 mL isolation buffer and placed in a magnetic separator (DynaMag-2 magnet; Thermo Fisher Scientific). The supernatant was aspired and the procedure was repeated this time using 0.5 mL isolation buffer. Following the aspiration of the supernatant, samples were resuspended in 50 µL isolation buffer and stored at −80 °C.

### 4.3. Relative Quantification of microRNA Expression by RT-qRT-PCR

Total RNA was isolated from exosome samples according to the manufacturer’s instructions using the miRNeasy Micro Kit (Qiagen, Hilden, Germany). *Caenorhabditis elegans*-miR-39 (cel-miR-39, miRNeasy Serum/Plasma Spike-In Control; Qiagen) was spiked into samples of serum exosomes and EpCAM-positive serum exosomes. CDNA was reverse transcribed from RNA eluates using the miScript II RT Kit (Qiagen) and preamplified using the miScriptPreAMP PCR Kit (Qiagen) according to the manufacturer’s standard protocol. For relative quantification of miRs, SYBR-Green based RT-qRT-PCR was performed as previously described using the miScript PCR system (Qiagen) [13]. Relative quantification of miR expression was analyzed using the 2^−ddCq^-method [59]. Cel-miR-39 was used as housekeeping control for serum exosomes and EpCAM-positive serum exosomes.

### 4.4. Western Blotting

Western blot analysis was used for detection of exosomal proteins. Exosome samples were dissociated solely in radioimmunoprecipitation buffer. Quantification of exosomal protein was conducted using the Pierce BCA Protein Kit (Thermo Fisher Scientific). Proteins were separated by sodium dodecyl sulfate (SDS) polyacrylamide gel electrophoresis and blotted on a blocked polyvinylidene fluoride membrane (Immobilon-P Transfer Membranes; Merck Millipore, Burlington, MA, USA). Primary antibodies were incubated overnight at 4 °C, and secondary antibodies were incubated for 1 h at room temperature. Primary antibodies were diluted at 1:400 (rabbit anti-CD63, sc-15363; Santa Cruz Biotechnology, Dallas, TX, USA) and at 1:1000 (mouse anti-ALIX, 2171; New England Biolabs, Ipswich, MA, USA). Horseradish peroxidase-linked secondary antibodies were diluted at 1:14,000 (anti-rabbit, A6154; Sigma-Aldrich) and at 1:130,000 (anti-mouse, A9044; Sigma-Aldrich). Peroxidase activity was detected using Immobilon Western Chemiluminescent HRP Substrate (Merck Millipore). ImageJ (Version 1.52, https://imagej.nih.gov/ij/) was used to determine intensity ratios in western blots.

### 4.5. Statistics

Statistical analysis was performed using IBM SPSS Statistics 23 (IBM Corp., Armonk, NY, USA), GraphPad Prism 7 (GraphPad Software, La Jolla, CA, USA), Microsoft Excel for Mac 16 (Microsoft Corp., Redmont, WA, USA), and R software (Version 3.6.1, https://www.r-project.org). Kruskal–Wallis test was used to test for significant differences in miR expression between multiple patient cohorts and the degree of linear correlation between two variables was stated as Pearson’s *r*. Fisher’s exact test was applied for comparison of clinicopathological parameters. Diagnostic potential of exosomal miR-200b and miR-200c was assessed by calculating the area under the ROC curve and its corresponding 95% CI. Log-rank test was applied to test for differences in OS and RFS between patient groups with high and low expression of miRs. MiR expression was classified as high and low according to the “surv_cutpoint” function implemented in the “survminer” R package. Postoperative survival was defined as the period of time between the date of surgery and the date of tumor relapse, tumor-related death, or last date of contact, whereas those patients still alive at the time of data cutoff were censored. A Cox proportional-hazards regression model was used to evaluate the prognostic value of miR-200b and miR-200c in total and EpCAM-positive serum exosomes. Variables that were significant in the univariate analysis were included in the multivariate analysis. Statistical significance was assumed at *p* ≤ 0.05.

## 5. Conclusions

Members of the miR-200-family have previously been attributed clinical utility as biomarkers in blood serum and tissue of patients with PDAC. Moreover, they have been shown to play an important role as modulators of EMT. In the present study, we could show that miR-200b and miR-200c derived from serum exosomes have great potential as diagnostic and prognostic liquid biopsy markers in PDAC patients. More specifically, we could identify miR-200b from EpCAM-positive serum exosomes as an independent prognostic factor for OS in PDAC.

## Figures and Tables

**Table 1:** Clinicopathologic data of all patients included in the study.

Category	Total	HC ^1^	CP ^2^	PDAC ^3^ UICC ^4^ II	PDAC UICC III	PDAC UICC IV	*p*-Value
***n*^5^**	89	22	11	18	22	16	
**Age (years)**							0.831
Median (range)	66 (26–87)	68 (43–87)	62 (55–80)	67 (53–82)	70 (48–82)	64 (26–78)	
<60	27	5	3	5	8	6	
≥60	62	17	8	13	14	10	
**Gender**							0.826
Female	36	11	5	6	8	6	
Male	53	11	6	12	14	10	
**Body mass index (kg/m^2^)**							0.205
Median (range)	25.0 (19.0–47.6)	27.0 (21.6–36)	26.0 (19.8–44.3)	24.0 (19.0–32.0)	24.8 (19.0–33.0)	24.0 (17.1–47.6)	
<25	43	6	5	10	12	10	
≥25	46	16	6	8	10	6	
**Smoking**							0.916
No	58	15	7	10	15	11	
Yes	31	7	4	8	7	5	
**Alcohol**							0.226
No	80	22	9	15	19	15	
Yes	9	0	2	3	3	1	
**Pre-surgical diabetes mellitus**							0.760
No	64	18	8	13	14	11	
Yes	25	4	3	5	8	5	
**Pre-surgical pancreatitis**							**<0.001**
No	68	22	0	16	15	15	
Yes	21	0	11	2	7	1	
**Pre-surgical CA.19-9 ^6^ (U/mL)**							**0.007**
Median (range)	142 (0.6–20640)	7.7 (3.5–18.9)	33.6 (10.0–218)	81.5 (0.6–3136)	238.2 (2.6–19160)	530.5 (2.4–20640)	
<30	21	5	4	4	5	3	
≥30	47	1	4	13	17	13	
**Pre-surgical CEA ^7^ (ng/mL)**							0.257
Median (range)	2.4 (0.2–54.8)	1.2 (0.2–3.1)	2.2 (0.2–4.5)	2.4 (0.4–9.6)	2.6 (0.2–54.8)	3.5 (0.6–14.2)	
<5	48	5	8	12	14	9	
≥5	14	0	0	3	7	4	
**Pre-surgical bilirubin (mg/dL)**							0.096
Median (range)	0.7 (0.2–15.9)	0.6 (0.2–1.6)	0.5 (0.2–1.4)	0.9 (0.2–5.5)	0.9 (0.3–7.1)	0.75 (0.3–15.9)	
<1.2	63	19	9	10	14	11	
≥1.2	23	2	1	7	8	5	

Bold values indicate significance (*p* ≤ 0.05, Fisher’s exact test). ^1^ HC, healthy controls; ^2^ CP, chronic pancreatitis; ^3^ PDAC, pancreatic ductal adenocarcinoma; ^4^ UICC, Union for International Cancer Control; ^5^ *n*, number of patients; ^6^ CA.19-9, carbohydrate antigen 19-9; ^7^ CEA, carcinoembryonic antigen.

**Table 2:** Survival analysis of histopathologic data of patients with PDAC UICC stage II to IV.

Category	Number of PDAC Patients	Predicted Median OS ^1^ (Months)	95% CI ^2^	*p*-Value
**Total**	56	13	7.9–18.1	
**Age (years)**				0.330
<60	19	11	5.8–16.2	
≥60	37	14	7.1–20.9	
**Gender**				0.895
Female	20	17	6.9–27.1	
Male	36	13	9.1–16.9	
**Body mass index**				0.542
<25	33	13	7.4–18.6	
≥25	23	14	9.2–18.8	
**Smoking**				0.905
No	36	14	5.9–22.1	
Yes	20	11	4.4–17.6	
**Alcohol**				0.621
No	49	13	6.5–19.5	
Yes	7	4	1.9–6.1	
**Pre-surgical diabetes mellitus**				0.128
No	38	17	12.6–21.4	
Yes	18	10	6.5–13.5	
**Pre-surgical pancreatitis**				0.994
No	46	13	7.2–18.8	
Yes	10	12	2.2–21.8	
**Pre-surgical CA.19-9 (U/L)**				0.600
<30	12	18	5.7–30.3	
≥30	43	13	8.6–17.4	
**Pre-surgical CEA (ng/mL)**				0.960
<5	35	18	10.9–25.1	
≥5	14	17	10.7–23.3	
**Pre-surgical bilirubin (mg/dL)**				0.984
<1.2	35	13	4.7–21.3	
≥1.2	20	14	7.7–20.3	
**UICC stage**				**0.013**
IIA	4	NR ^3^		
IIB	14	18		
III	22	17	8.7–25.3	
IV	16	8	5.8–10.2	
**T stage**				0.062
T1	1	NR		
T2	5	NR		
T3	22	14	4.2–23.8	
T4	24	11	4.6–17.4	
**Nodal invasion**				0.373
N0	9	34		
N1	23	12	5.5–18.5	
N2	6	10		
**Metastasis**				**0.008**
M0	40	18	11.0–25.0	
M1	16	8	5.8–10.2	
**Type of surgery**				**0.006**
PPPD ^4^	15	NR		
Pancreatic left resection	9	18		
Excisional biopsy	32	11	6.6–15.4	
**Administration of chemotherapy**				**<0.001**
No	9	4		
Yes	46	14	7.2–20.8	

Bold values indicate significance (*p* ≤ 0.05, log-rank test). ^1^ OS, overall survival; ^2^ CI, confidence interval; ^3^ NR, not reached; ^4^ PPPD, pylorus-preserving pandreaticoduodenectomy.

**Table 3:** Univariate and multivariate survival analysis for overall survival of PDAC patients.

Variable	Subset	Univariate Analysis		Multivariate Analysis	
		HR (95% CI) ^1,2^	*p*	HR (95% CI)	*p*
Age (years)	≥60/<60	0.70 (0.34–1.50)	0.340		
Gender	Male/Female	1.05 (0.50–2.23)	0.897		
Body mass index	≥25/<25	0.71 (0.44–1.52)	0.380		
Smoker	Yes/No	0.95 (0.43–2.10)	0.906		
Alcohol	Yes/No	1.31 (0.44–3.91)	0.626		
Pre-surgical diabetes	Yes/No	1.76 (0.83–3.73)	0.139		
Pre-surgical pancreatitis	Yes/No	1.00 (0.29–3.37)	0.994		
Pre-surgical CA.19-9 (U/L)	≥30/<30	1.25 (0.53–2.95)	0.607		
Pre-surgical CEA (ng/mL)	≥5/<5	0.98 (0.36–2.65)	0.960		
Pre-surgical bilirubin (mg/dL)	≥1.2/<1.2	1.01 (0.45–2.27)	0.984		
UICC stage	III-IV/II	3.27 (1.33–8.05)	**0.010**	2.97 (1.00–8.88)	0.051
T stage	T3-4/T1-2	5.00 (0.40–63.1)	0.214		
Nodal invasion	N1-2/N0	1.71 (0.67–4.38)	0.263		
Metastasis	M1/M0	2.63 (1.23–5.60)	**0.013**	2.11 (0.85–5.24)	0.109
Grading	G3/G2	2.00 (0.59–6.85)	0.268		
Lymphatic invasion	L1/L0	0.96 (0.23–4.06)	0.956		
Vene invasion	V1/V0	2.40 (0.57–10.2)	0.235		
Resection margin	R1/R0	1.61 (0.35–7.31)	0.540		
Surgery	PPPD ^3^/left	4.32 (0.61–30.7)	0.144		
Chemotherapy	Yes/No	0.09 (0.02–0.43)	**0.002**	0.05 (0.01–0.31)	**0.001**
miR-200b (EpCAM-Exo ^4^)	High/Low	2.23 (1.04–4.76)	**0.040**	2.40 (1.03–5.58)	**0.044**
miR-200c (S-Exo ^5^)	High/Low	2.10 (1.01–4.37)	**0.046**	0.92 (0.40–2.14)	0.924

Bold values indicate significance (*p* ≤ 0.05). ^1^ HR, hazard ratio; ^2^ CI, confidence interval; ^3^ PPPD, pylorus-preserving pancreaticoduodenectomy; ^4^ EpCAM-Exo, EpCAM-positive serum exosomes; ^5^ S-Exo, total serum exosomes.

## Supplementary Materials

The following are available online at https://www.mdpi.com/2072-6694/12/1/197/s1: Table S1: Distribution of histopathologic characteristics of PDAC patients across UICC tumor stages; Table S2: Differential expression of miR-200b and miR-200c in serum exosomes; Figure S1: Whole western blots; Figure S2: ROC curve analysis of miR panel + CA.19-9 to differentiate between UICC tumor stages.
