# Supplementary material for: Potential of Exosomal microRNA-200b as Liquid Biopsy Marker in Pancreatic Ductal Adenocarcinoma
Source: Cancers (Basel). 2020 Jan 13;12(1):197. doi: 10.3390/cancers12010197 (PMC7016821; doi:10.3390/cancers12010197)
Supplement: Supplementary file 1 [file cancers-12-00197-s001.pdf]

Table S1. Distribution of histopathologic characteristics of PDAC patients across UICC tumor stages.

	**Total**	**PDAC UICC IIA**	**PDAC UICC IIB**	**PDAC UICC III**	**PDAC UICC IV**
**n** [1]	56	4	14	22	16
**T stage**					
T1	1	0	1	0	0
T2	5	0	3	2	0
T3	22	4	10	3	5
T4	24	*N/A*	*N/A*	17	7
Tx	4	0	0	0	4
**Nodal invasion**					
N0	9	4	*N/A*	5	0
N1	23	*N/A*	14	5	4
N2	6	*N/A*	*N/A*	6	0
Nx	18	0	0	6	12
**Metastasis**					
M0	40	4	14	22	N/A
M1	16	N/A	N/A	N/A	16
**Grading**					
G2	16	2	9	4	1
G3	12	2	4	4	2
Gx	28	0	1	14	13
**Lymphatic invasion**					
L0	16	3	10	3	0
L1	9	1	4	3	1
Lx	31	0	0	16	15
**Vene invasion**					
V0	20	4	12	4	0
V1	6	0	2	2	2
Vx	30	0	0	16	14
**Perineural invasion**					
Pn0	3	2	1	0	0
Pn1	20	2	12	5	1
Pnx	33	0	1	17	15

[1] n, number of patients.

Table S2. Differential expression of miR-200b and miR-200c in serum exosomes.

	CP	UICC II	UICC III	UICC IV	UICC II-IV	UICC II	UICC III	UICC IV	UICC II-IV
	vs. healthy controls					*vs. CP*			
**miR-200b in total serum exosomes**									
$2^{-\Delta\Delta Cq}$ [1]	1.08	2.57	5.10	4.99	4.04	2.38	4.72	4.61	3.73
p [2]	0.857	**0.037**	**<0.001**	**0.001**	**<0.001**	**0.001**	**0.002**	**0.011**	**0.005**
r [3]	0.03	0.33	0.61	0.53	0.45	0.29	0.55	0.50	0.35
**miR-200b in EpCAM-positive serum exosomes**									
$2^{-\Delta\Delta Cq}$	1.02	2.38	3.11	1.91	2.48	2.34	3.05	1.87	2.44
p	0.775	**0.020**	**0.013**	0.156	**0.008**	0.097	0.080	0.357	0.089
r	0.05	0.37	0.38	0.23	0.30	0.31	0.31	0.18	0.21
**miR-200c in total serum exosomes**									
$2^{-\Delta\Delta Cq}$	1.12	1.44	2.02	2.55	1.92	1.28	1.80	2.28	1.72
p	0.735	0.235	**0.046**	**0.022**	**0.024**	0.523	0.208	0.114	0.190
r	0.06	0.18	0.31	0.38	0.26	0.12	0.22	0.32	0.16

Bold values indicate significance ($p \leq 0.05$, Kruskal-Wallis test). [1] $2^{-\Delta\Delta Cq}$, miR expression relative to healthy controls or chronic pancreatitis; [2] *p*, *p*-value; [3] *r*, Pearson correlation coefficient

Figure S1. Whole western blots & intensity ratios relative to healthy control. Red boxes indicate cropped area.

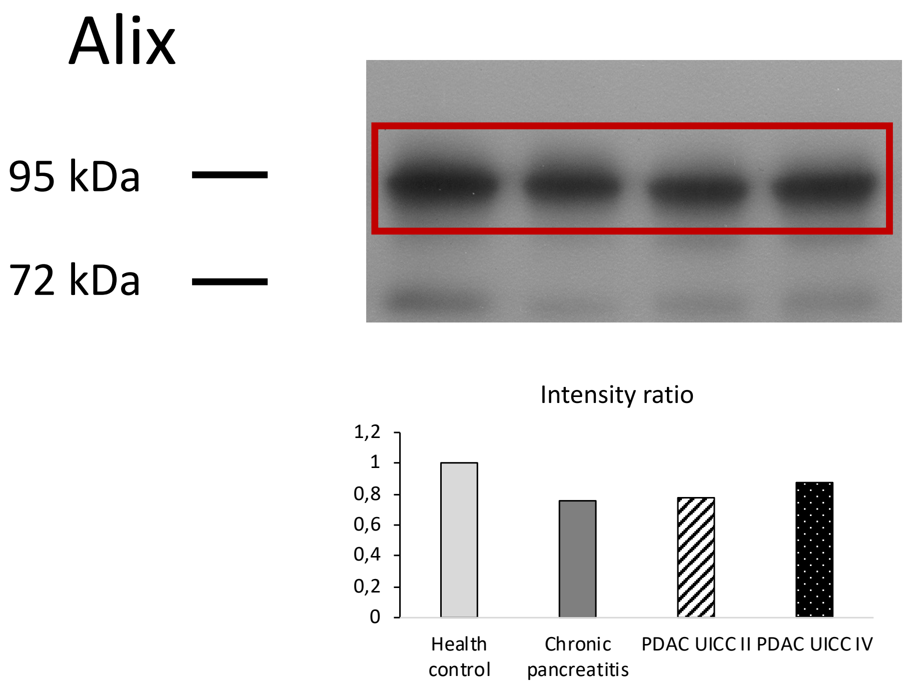

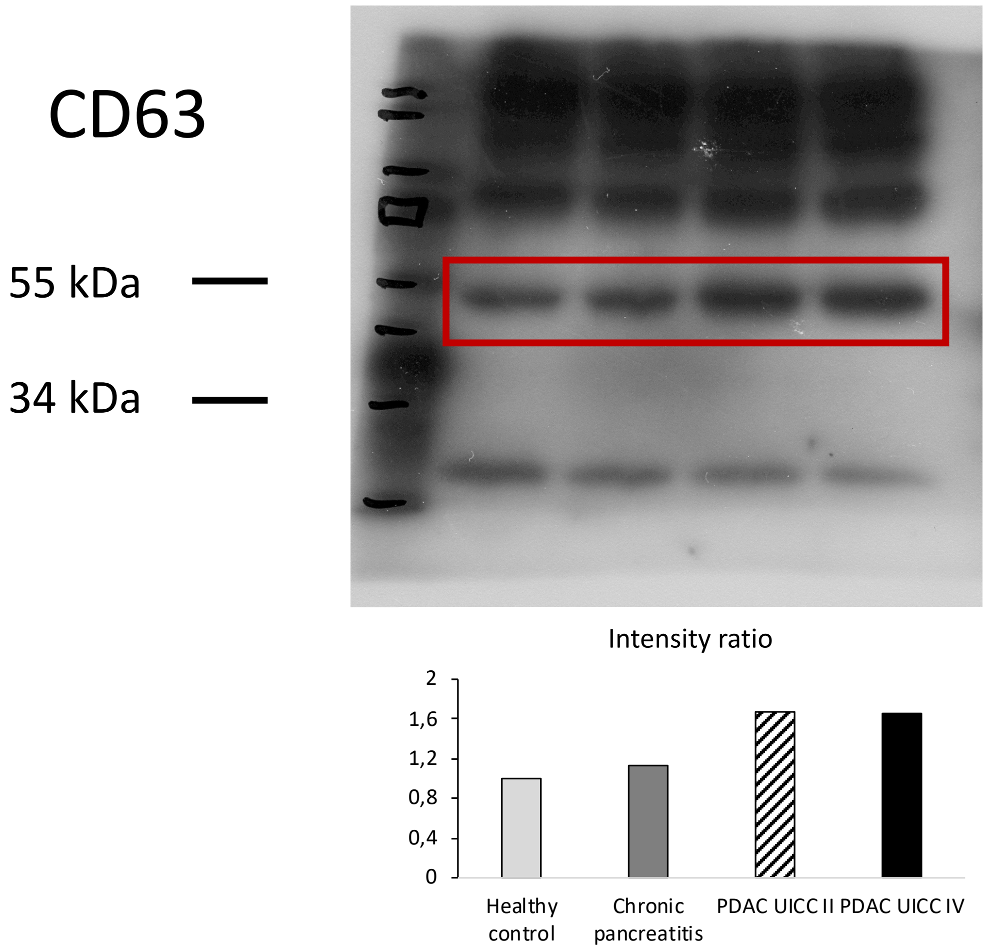

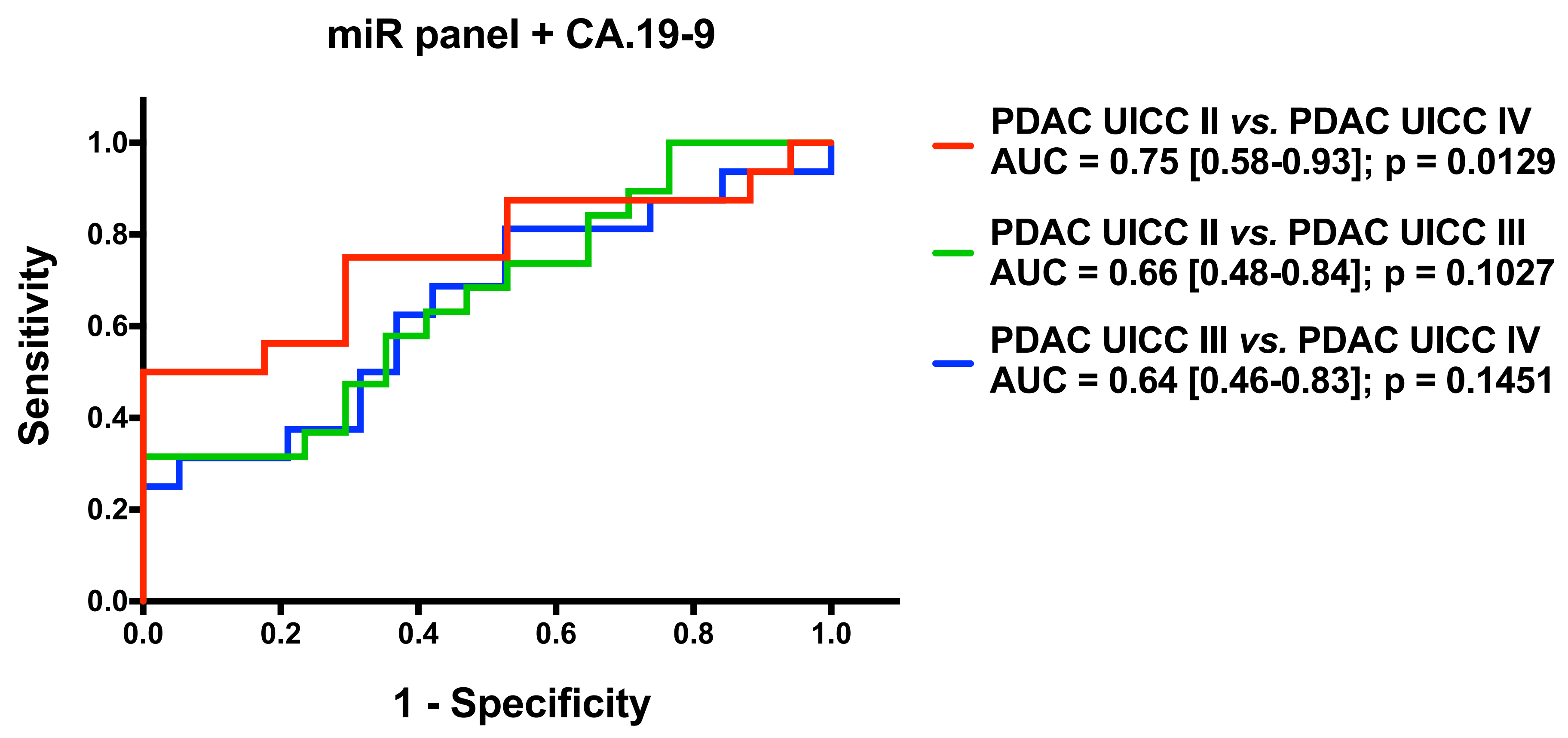
miR panel + CA.19-9
Sensitivity
1 - Specificity
0.0
0.2
0.4
0.6
0.8
1.0
PDAC UICC II *vs.* PDAC UICC IV
AUC = 0.75 [0.58-0.93]; p = 0.0129
PDAC UICC II *vs.* PDAC UICC III
AUC = 0.66 [0.48-0.84]; p = 0.1027
PDAC UICC III *vs.* PDAC UICC IV
AUC = 0.64 [0.46-0.83]; p = 0.1451
